# Successful radiation treatment of anaplastic thyroid carcinoma metastatic to the right cardiac atrium and ventricle in a pacemaker-dependent patient

**DOI:** 10.1186/1748-717X-6-16

**Published:** 2011-02-14

**Authors:** Tina Dasgupta, Igor J Barani, Mack Roach

**Affiliations:** 1 From the Department of Radiation Oncology, 1600 Divisadero Street, Suite H1031, San Francisco, California - 94102-1708, USA

## Abstract

Anaplastic thyroid carcinoma (ATC) is a rare, aggressive malignancy, which is known to metastasize to the heart. We report a case of a patient with ATC with metastatic involvement of the pacemaker leads within the right atrium and right ventricle. The patient survived external beam radiation treatment to his heart, with a radiographic response to treatment. Cardiac metastases are usually reported on autopsy; to our knowledge, this is the first report of the successful treatment of cardiac metastases encasing the leads of a pacemaker, and of cardiac metastases from ATCs, with a review of the pertinent literature.

## Background

Anaplastic thyroid carcinoma (ATC) is a rare, aggressive malignancy with a median survival of 6 months. Distant metastases - usually to lungs and bone - present early in the course of disease[1]. ATC is also one of the few cancers known to metastasize to the heart [2].

Secondary cardiac tumors are usually reported at autopsy, and can involve various anatomic structures of the heart. While cardiac metastases can be treated with external beam radiation, cardiac toxicity remains dose-limiting and must be taken into consideration during radiation treatment planning for patients with poor cardiac function and pacemaker dependence.

We report the case of a patient with ATC who presented with intraventricular metastases encasing the electrical leads of his pacemaker. After a course of palliative radiation therapy to his right atrium and ventricle, the patient survived to demonstrate radiographic response to treatment.

As cardiac metastases are increasing in incidence, we detail the radiation methods used to treat these intracardiac metastases, including specific precautions taken for the pacemaker leads within the field of radiation. To our knowledge, this is the first report of the successful treatment of cardiac metastases from from ATCs, and of mural metastases encasing the leads of a pacemaker.

## Case

The patient is an 80 year old male with a past medical history of atrial fibrillation with sinus block with dual chamber pacemaker placed in November 2006, and a complicated oncologic history including breast cancer in the 1970s treated with left-sided mastectomy and axillary lymph node dissection; prostate cancer treated with intensity modulated radiation therapy (IMRT) in 2001; mucosal melanoma with metastases to small bowel treated with small bowel resection in 2005; and multiple skin cancers. He was treated with a total thyroidectomy for anaplastic thyroid carcinoma in March 2008, followed by post-operative cisplatin-based chemo-radiation therapy to the surgical bed and the draining lymph nodes. A subsequent left lung nodule was treated with thoracotomy and wedge resection in December 2008, with documented metastatic anaplastic thyroid carcinoma on pathology. He also received one cycle of Abraxane and Bevacizumab in February 2009.

The patient had been asymptomatic and in his usual state of health until July 2009 when he presented with a 2 month history of decreased exercise tolerance and orthostatic hypotension. Workup revealed a loss of atrial function, leaving the patient dependent on his pacemaker. An outpatient echocardiogram was concerning for "intracavitary irregular densities" in the right ventricle and right atrium. CT Chest with contrast revealed a 5.1 × 4.8 cm right atrial mass, with a broad base of attachment at the right atrial posterior wall and extension into both the inferior and superior vena cava. There was a notable displacement of pacemaker leads. The right ventricle also demonstrated an irregular lobulated 6.8 × 2.5 cm mass attached to the ventricular septum. Retrospective evaluation of a prior PET-CT from June 2009 confirmed increased FDG uptake within the right atrium and right ventricle.

In mid-July 2009, the patient was admitted to University of California San Francisco Moffitt Hospital for cardiac telemetry and management of this intracardiac mass. Admission labs showed thrombocytopenia with platelets ranging between 20 and 35. The differential diagnosis for right heart masses included metastases from anaplastic thyroid carcinoma or melanoma, a new primary cardiac malignancy, or a thrombus.

A Fibrinogen level was within normal limits, and hematology smears were negative for schistocytes. A bone marrow biopsy demonstrated a normocellular marrow for the patient's age with mixed trilineage hematopoesis and no evidence of lymphoma or thrombus. A trial of dexamethasone for suspected idiopathic thrombocytic purpura (ITP) did not impact the thrombocytopenia. The differential diagnosis for the thrombocytopenia therefore remained a consumptive coagulopathy secondary to tumor, versus tumor-associated immune thrombocytopenia.

After careful consideration at a multi-institutional tumor board, it was decided to treat these intracardiac metastases with radiation therapy. A pre-treatment electrophysiologic interrogation showed intermittent loss of capture by the pacemaker, most likely secondary to growth of the intracardiac mass. Therefore, a new pacemaker with epicardial leads was emergently placed. During this procedure, biopsy of the intracardiac mass was performed, confirming metastatic anaplastic thyroid carcinoma.

**Figure 1:**
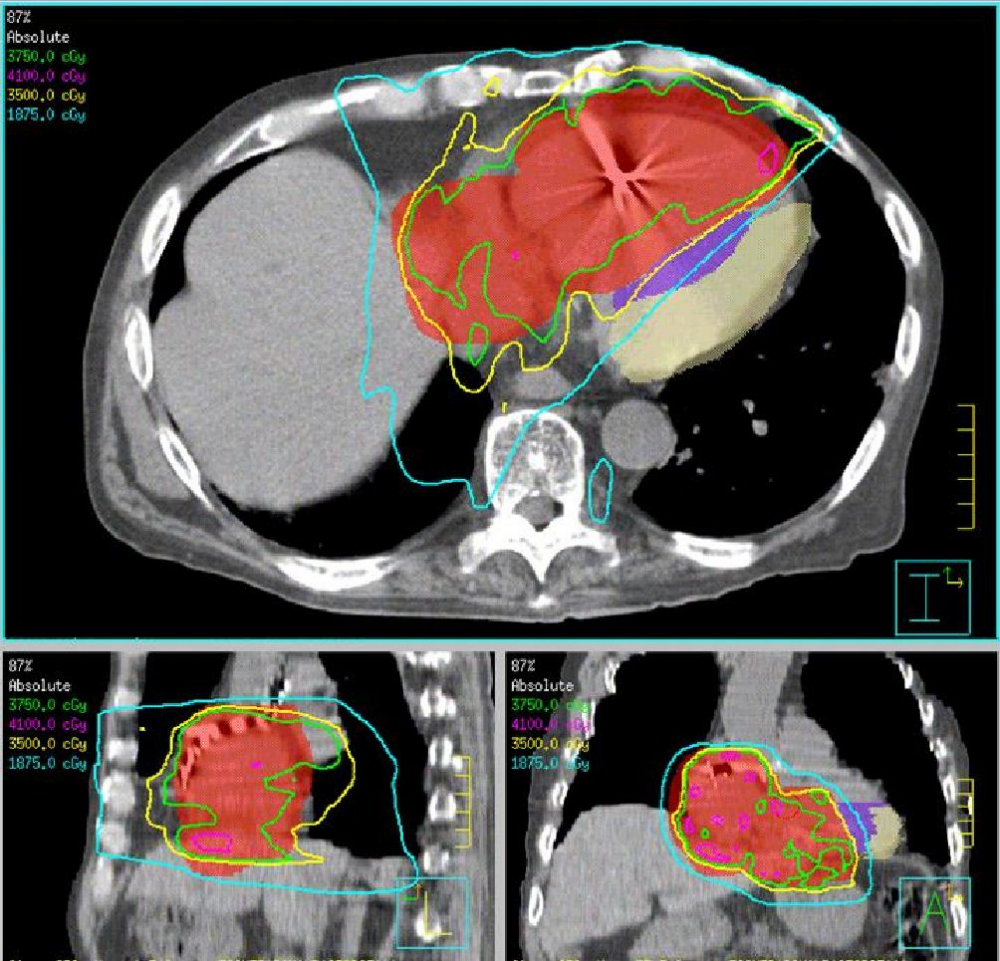
**Radiation treatment plan for patient with right atrial and ventricular metastases from anaplastic thyroid carcinoma**. The PTV is delineated in red and received 37.50 Gy in 15 fractions, prescribed to the 87% isodose line. The median left ventricle (purple) and lateral left ventricle (beige) were delineated as avoidance structures in this IMRT treatment plan. The 1875 cGy (18.7 Gy) isodose line is shown in light blue; the 3500 cGy (35 Gy) isodose line, in yellow; the 3750 cGy (37.5 Gy) isodose line, in green; and the 4100 cGy (41 Gy) isodose line, in pink.

Radiation therapy to the right atrium and part of the right ventricle was initiated at 2.5 Gy per fraction for 15 fractions to a total dose of 37.5 Gy, with an intended maximum dose in the tumor areas just exceeding 40 Gy (see below) (Figure 1). Paclitaxel (50 mg/m^2^) was administered concurrently on days 1 and 8 of radiation treatment.

During the course of his radiation treatment, the pacemaker demonstrated full capture. A single episode of ventricular undersensing with pacing stimuli during T-waves was successfully addressed by the reprogramming of the device. Transcutaneous pacer was available during treatment should failure of the primary pacing device occur. Echocardiograms during radiation treatment showed that the intracardiac mass had not increased in size. The patient required platelet transfusions approximately every 48 hours, and his platelet count held steadily around 18 to 20. Given his leukopenia and sepsis, Abraxane was withheld after two courses.

After discharge, the patient participated in regular activities of daily living, including work-related meetings and exercise on the treadmill, but experienced persistent dyspnea on exertion. His pacemaker continued to demonstrate full capture without evidence of dysfunction.

**Figure 2:**
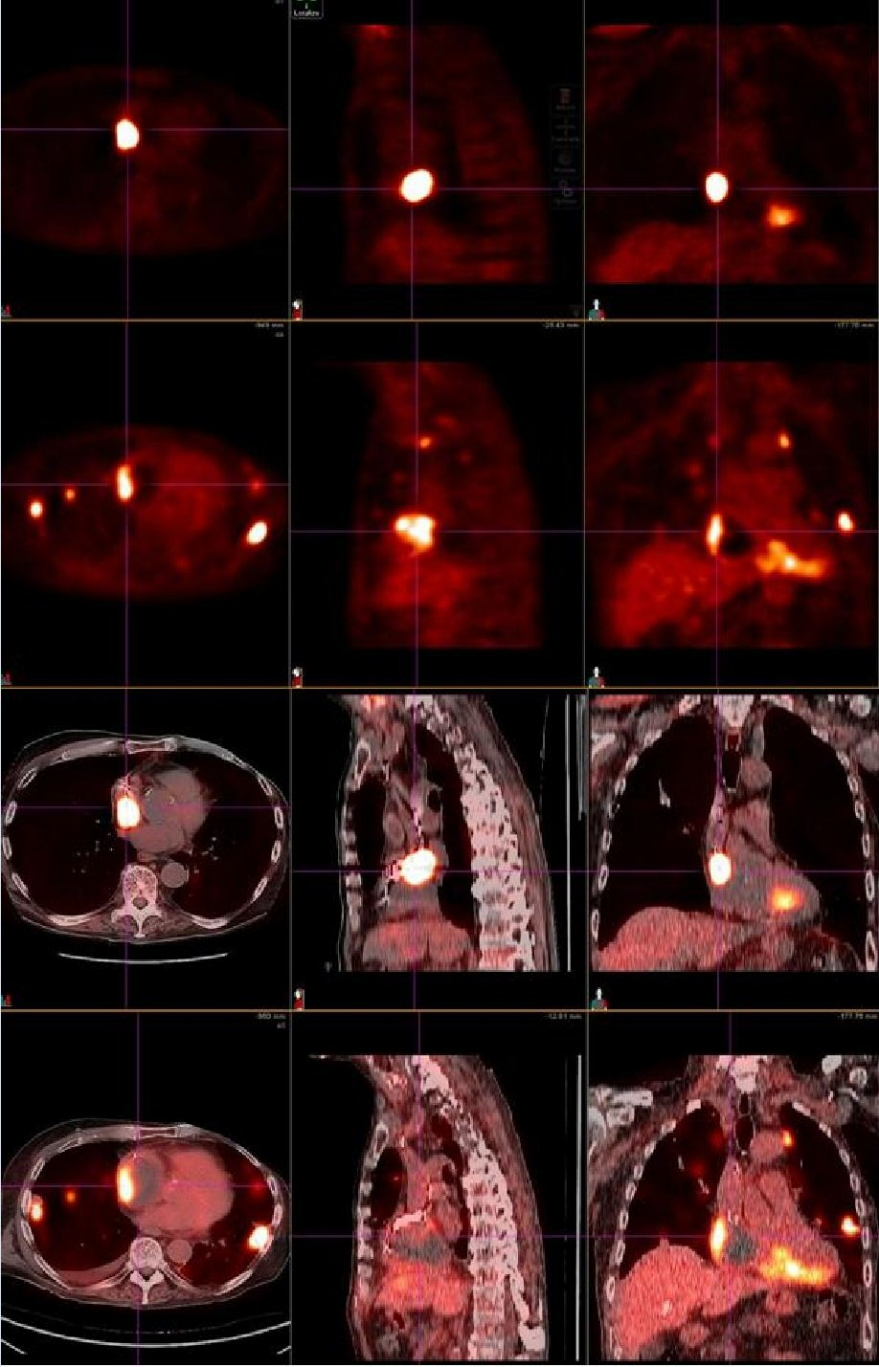
**PET Response to treatment of an intracardiac metastases in the R ventricle and atrium**. The top panel demonstrates a PET-CT scan of the hypermetabolic tumor mass prior to treatment on June 8 2009; the lower panel shows the tumor mass with notably decreased FDG uptake after treatment on August 30 2009.

In late August 2009, less than one month after completion of treatment, a PET-CT showed decreased FDG uptake right atrium (maximum SUV decreased from 27.9 to 7.8) and stable FDG uptake within the right ventricle (Figure 2). There was some questionable uptake in the interventricular septum, representing normal physiologic uptake or residual disease. Unfortunately, multiple pulmonary and chest wall metastases were subsequently detected.

The patient completed one additional course of palliative radiation therapy to a symptomatic left chest wall metastasis. He died in his home two months after completion of radiation therapy.

## Conclusions

### I. Secondary cardiac tumors are increasing in incidence, have various methods of spread and can affect any anatomic region of the heart

There have been several reports of cardiac metastases in the the literature [3,4]. Intracardiac metastases are reported from several different primary cancers, including melanoma, bladder[4], sarcoma[5], lung, lymphoma, breast carcinoma[6] and cervix [7]. Cardiac metastases from primary anaplastic thyroid carcinoma are rare - an autopsy series has reported the rate as 0 to 2% [2].

Different routes of cancer spread have been reported, and include hematogenous, lymphatic, and direct extension to the heart or thoracic duct [2,8]. Retrograde lymphatic spread is predominant. The majority of lymphatic ducts of the heart are located on the pericardial surface, where they coalesce adjacent to the aortic root; obstruction of these channels leading to malignant pericardial effusions [8]. The coronary arteries are the primary conduits of hematogenous spread. As metastatic cancer cells are filtered in the hepatic and pulmonary circulation, metastases are unlikely to reach cardiac tissue without metastatic disease in other organs [8].

Metastatic lesions may involve any anatomic region of the heart, including most commonly the epicardium and pericardium. Metastases within the cardiac chambers are rare [6,9,10]. In primary anaplastic thyroid carcinoma, cardiac metastases have been reported in the myocardium and pericardium, as well as within the ventricles [2].

### II. Secondary cardiac tumors have been successfully treated with external beam radiation

Various cardiac tumors have been reported to respond to radiation. These include a primary cardiac sarcoma involving right ventricular outflow track treated with a dose 6300-7400 rads delivered with 4 fields in the 1960's. The patient remained asymptomatic for 8 months, and autopsy identified no residual cardiac tumor[11]. Cases of leukemic infiltration of the myocardium presenting with arrhythmia [12], and interventricular septum metastases with malignant pleural effusion responding to radiation have also been reported [13]. In a case of cardiac metastases from cervical carcinoma, one patient with right ventricular and intraventricular septum metastases was treated with chemoradiation (2/60 Gy with concurrent 5-fluorouracil and cisplatin) and survived 7 months after presentation[10]. A recent report also demonstrated response of intraventricular metastases from small cell lung cancer to chemotherapy and radiation (carboplatin and etoposide followed by IMRT to 60 Gy to the lung mass, mediastinal LNs and cardiac metastases). Two months after treatment, follow-up PET CT showed no residual uptake in the R ventricle or mediastinal LNs, but persistent uptake in the lungs [14].

From these case studies, no consensus on the dose required to control a secondary cardiac tumor can be established. In radiosensitive tumors like lymphoma, doses of even 20 Gy may be sufficient. However, more radioresistant tumors may require higher doses like 45 Gy with a possible additional boost of 10 to 15 Gy for adequate control[3,15].

### III. Cardiac toxicity is dose-limiting in the treatment of cardiac metastases with external beam radiation

The dose tolerance of the pericardium is limiting[10], as the most common manifestation of radiation-induced heart disease (RIHD) is late-onset, chronic pericardial disease[9]. However, any other anatomic region of the heart can manifest cardiac damage secondary to radiation, including electrical conduction system, coronary arteries, cardiac valves, myocardium and endocardium [9,16]. Therefore, while the dose tolerance of the whole heart - 60 Gy if 25% of the heart is irradiated, and 45 Gy if 65% of the heart volume is irradiated [17] - is usually taken into consideration, the different anatomic subsites must be considered. Risk of RIHD increases with doses > 40 Gy over 4 weeks for pericardial disease, > 35-60 Gy for myocardial disease, > 30 Gy for valvular disease, and > 30 Gy for coronary artery disease (especially in younger patients, with concomitant chemotherapy), though doses as low as 5 Gy have been associated with increased risk of coronary artery disease[9,17]. Beam energy, dose per fraction [18,19], concurrent chemotherapy - especially with anthracyclines[16] - affect cardiac toxicity from radiation. Most patients experiencing severe complications had 60% or more of their cardiac silhouette irradiated, and risk of RIHD ranged from 6.6 to 29%. Preclinical trials suggest that cardiac gating [20] may reduce the incidence of RIHD.

RIHD is additionally divided into early and late toxicity. Early toxicity, presenting within 2 to 6 months, is most frequently pericarditis [21-23]. Radiation induced valvular disease manifests within ten to fifteen years of irradiation and management is similar to other types of valvular disease[9]. In younger patients who have received mediastinal irradiation for either Hodgkin's lymphoma or breast cancer (even if the heart waspurposefully blocked or omitted from the treatment portals [24]), late toxicity is typically manifested as early or aggravated coronary artery disease [19,25-32]. However, modern radiation methods have reduced incidences of both acute and late pericardial toxicity [9]. The patient in this case report experienced no acute toxicity secondary to radiation.

### IV. The radiation of cardiac metastases encasing pacemaker leads has not been previously reported

Until 1978, pacemakers based on bipolar technology were commonly used, and capable of withstanding cumulative radiation doses of up to 300 Gy without dysfunction. However, the metal oxide semiconductors of modern pacemakers render them more sensitive to radiation[33]. There are only nine reported cases of radiation-related pacemaker malfunction in the literature since 1983[33], with widely varying doses of radiation observed to cause pacemaker dysfunction[34,35]. In one study, 6% of devices showed dysfunction at doses below 2 Gy [35]. In another study, most devices tolerated a cumulative dose of more than 90 Gy before failing, and only of nineteen studied devices failed with a cumulative dose of 20 Gy [34]. Either direct or indirect damage to the circuit (if the pacemaker is out of the treatment field) by the electromagnetic field is the proposed mechanism of damage. Therefore, while there is no consensus for a safe threshold of radiation for the pacemaker within a treatment field, the modern pacemaker seems relatively resistant to radiation-related malfunction[34].

For our case subject, the cumulative mean dose to the pacemaker was 0.2615 Gy with a maximum dose 0.37 Gy. TLDs (thermoluminescent dosimeters) placed during the first fraction showed the effective dose to the superior aspect of the pacemaker was (0.862 +/- 0.104 Gy). The treatment plan was modified to ensure that the cumulative dose to the device itself was less than 0.50 Gy (0.461 Gy +/- 0.41 Gy). Because of the theoretical possibility that treatment response of the tumor encasing the pacemaker leads could result in a loss of electrical capture, the pacemaker was interrogated before and upon completion of every treatment. The irradiation was carried out under continuous EKG monitoring via transcutaneous pads which could also be used for external pacing in case of the primary pacer failure or loss of primary capture.

### V. Radiation therapy can successfully palliate cardiac metastases while preserving quality of life

We decided to treat this patient with IMRT to limit the cardiac dose and result in less cardiac toxicity. The patient was treated in a supine position immobilized with a wing board. A CT simulation with 4D respiratory gating in 8 phases of respiration was acquired in both free-breathing and breath hold positions, with 1.55 mm slice reconstruction and no contrast. The Gross Tumor Volume (GTV) was defined as the right atrial and right ventricular tumor. An Internal Tumor Volume (ITV) (with an additional 1 to 1.5 cm cardiac margin) was generated from the Maxiumum Intensity Projection (MIP) using the CT simulation data from all 8 phases of respiration, taking into account cardiac motion of the tumor. A Planning Target Volume (PTV) was defined as the ITV with an additional 5 mm margin in all dimensions. In the free-breathing CT scan, which was used for the final treatment planning, the lateral left ventricle and medial left ventricle (including the interventricular septum) were contoured as avoidance structures. The patient was treated with an IMRT plan 2.5 Gy per fraction to 87% isodose line delivered in 15 fractions with 8 coplanar beams and 6 mV photons. The actual maximum dose to the tumor from all beams was 43.1 Gy (Figure 1). The patient had previously received radiation to the mediastinum, and his prior treatment plan was dearchived and reconstructed to avoid overlap of radiation fields and minimize the dose to the lungs. Dosimetric paramters are outlined in Table 1.

**Table 1:** Dosimetric parameters of radiation treamtment plan

Structure	Parameter	Percentage of tissue receiving threshold dose
R lung	V20	9.7%
L lung	V20	1.95%
GTV	V37.5	95.1%
PTV	V37.5	69.77%
Lateral Left Ventricle	V37.5	0.01%
Lateral Left Ventricle	V25	6.14%
Medial Left Ventricle	V37.5	0.95%
Medial Left Ventricle	V25	45.7%

Abbreviations: V20 = percentage of tissue receiving ≥ 20 Gy; V37.5 = percentage of tissue receiving ≥ 37.5 Gy; V25 = percentage of tissue receiving ≥ 25 Gy

After completing treatment, the patient did not experience any significant acute toxicities from the treatment. He remained exceptionally active until his death.

### VI. Case Summary and Recommendations

We report a successful PET-proven response to IMRT of a secondary cardiac tumor, which encased the leads of a dual-chamber pacemaker in a pacer-dependent patient. The treatment plan was designed to prevent overlap with the patient's prior mediastinal radiation fields, and also to minimize toxicity to the whole heart and left ventricle. This treatment was well-tolerated by the patient, with preservation of quality of life.

In their review of secondary cardiac tumors, Cham et al. suggest that "cardiac metastases should be strongly suspected in the cancer patient with sudden onset of unexplained tachycardia, arrhythmia, or congestive heart failure"[36]. In the aging US population where the use of permanent pacemakers is increasing[37], cancer patients with pacemakers will only become more common. We recommend evaluation for cardiac metastases in patients with disseminated disease who experience symptoms of unexplained tachyarrhythmias or other cardiac abnormalities. Proper cardiac evaluation may be warranted in these high-risk patients. Traditional oncologic staging techniques are generally not adequate for proper evaluation. For example, the naturally high FDG uptake of the cardiac muscle on a staging PET/CT scan may obscure cardiac metastases. As demonstrated by our case subject, cardiac metastases can be effectively palliated with radiation therapy to meaningful doses with limited toxicity using modern techniques. Each case should be individually evaluated for palliative treatment giving full consideration to the patient's and family's goals of care.

## List of Abbreviations

The following abbreviations have been used in this manuscript: 5FU: 5-fluorouracil; ATC: anaplastic thyroid carcinoma; AED: automated external defibrillator; FDG: fluorodeoxy glucose; GTV: gross tumor volume; IMRT: intensity modulated radiation therapy; ITP: idiopathic thrombocytic purpura; ITV: integrated target volume; PET-CT: positron emission tomography computed tomography; PTV: planning target volume; RIHD: radiation induced heart disease; TLD: thermoluminscent dosimetry; V20: percentage of tissue receiving ≥ 20 Gy; V37.5: percentage of tissue receiving ≥ 37.5 Gy; V25: percentage of tissue receiving ≥ 25 Gy.

## Consent

Written informed consent was obtained from the patient for publication of this case report and any accompanying images. A copy of the written consent is available for review by the Editor-in-Chief of this journal.

## Competing interests

The authors declare that they have no competing interests.

## Authors' contributions

TD, IJB and MR III were the radiation oncologists involved in caring for the patient discussed in this the case report. They designed and delivered the radiation treatment plan described above. All authors read and approved the final manuscript.
